# Microbial Community, Newly Sequestered Soil Organic Carbon, and δ^15^N Variations Driven by Tree Roots

**DOI:** 10.3389/fmicb.2020.00314

**Published:** 2020-02-27

**Authors:** Wenchen Song, Xiaojuan Tong, Yanhong Liu, Weike Li

**Affiliations:** ^1^College of Forestry, Beijing Forestry University, Beijing, China; ^2^Institute of Microbiology, Chinese Academy of Sciences, Beijing, China; ^3^Key Laboratory for Forest Resources and Ecosystem Processes of Beijing, Beijing Forestry University, Beijing, China

**Keywords:** tree roots, microbial community, carbon sequestration, ^15^N isotopic tracing, ^13^C isotopic tracing, plant–soil interactions

## Abstract

Rhizosphere microbes in forests are key elements of the carbon sequestration of terrestrial ecosystems. To date, little is known about how the diversity and species interactions of the active rhizomicrobial community change during soil carbon sequestration and what interactions drive these changes. In this study, we used a combination of DNA and stable isotope method to explore correlations between the composition of microbial communities, N transformation, and the sequestration *de novo* of carbon in soils around *Pinus tabuliformis* and *Quercus variabilis* roots in North China. Rhizosphere soils from degraded lands, primary stage land (tree roots had colonized in degraded soil for 1 year), and nature forest were sampled for analyses. The results showed that microbial communities and newly sequestered soil organic carbon (SOC) contents changed with different tree species, environments, and successive stages. The fungal unweighted and weighted UniFrac distances could better show the different microbial species structures and differences in successive stages. Newly sequestered SOC was positively correlated with the bacterial order *Rhizobiales* (in *P. tabuliformis* forests), the fungal order Russulales (in *Q. variabilis* forests), and δ^15^N. Consequently, the bacterial order *Rhizobiales* acted as an important taxa for *P. tabuliformis* root-driven carbon sequestration, and the fungal order Russulales acted as an important taxa for *Q. variabilis* root-driven carbon sequestration. The two plant species allocated root exudates to different portion of their root systems, which in turn altered microbial community composition and function. The δ^15^N of soil organic matter could be an important indicator to estimate root-driven carbon sequestration.

## Introduction

Root-driven carbon is a major flux in the terrestrial carbon cycle and is crucial for carbon sequestration, soil quality, and ecosystem function (Pausch and Kuzyakov, 2017). Recent studies have found that soil organic carbon (SOC) is mainly derived from roots and rhizomicroorganism interactions (Treseder and Holden, 2013; Cheng et al., 2014). Roots can greatly affect soil carbon sequestration by accelerating or decelerating the turnover rate of SOC (Kuzyakov, 2010), transferring organic carbon directly from the plant to the underground carbon pool (Rillig and Mummey, 2006; Tefs and Gleixner, 2012; Keymer et al., 2017), and modulating SOC from biomass and SOC secretion by microorganisms (Clemmensen et al., 2013). Environmental factors affecting the carbon sequestration capacity of roots mainly include soil physical and chemical properties, such as moisture, pH, temperature, atmospheric CO_2_ concentration, and nitrogen and phosphorus contents (Rukshana et al., 2013; Cheng et al., 2014; Song et al., 2018). Moreover, rhizomicroorganisms affect the decomposition and sequestration of SOC through the acquisition of nutrients by soil microorganisms (Pausch and Kuzyakov, 2017). Competition and symbiosis between roots and soil microorganisms regulate SOC sequestration (Kuzyakov and Xu, 2013), and soil microbes are known to be strongly involved in plant community succession and to influence carbon sequestration (Bardgett and van der Putten, 2014; Morriën et al., 2017). However, although the different mechanisms involved in these processes have been studied separately by soil scientists and ecologists, these mechanisms should be studied together because they are related (Dignac et al., 2017). Further work is needed to experimentally test the responses of microbial species to plant root exudates and their potential for use in speeding up nature restoration (Wubs et al., 2016).

Forests are the largest carbon sink in the world, accounting for about two-thirds of the total carbon sequestration in terrestrial ecosystems (Pan et al., 2011). Tree roots can effectively transport photosynthetic organic matter to deeper underground (Tefs and Gleixner, 2012) and make rhizosphere microorganisms more active at a soil depth of 0–100 cm (Song et al., 2016), thus affecting the size and composition of the whole soil carbon pool (Callesen et al., 2016). China holds the largest plantation area in the world, and planted forests may therefore play an important role in sequestrating atmospheric CO_2_ (Guo et al., 2013). However, there is no consensus on whether and how much can forests sequestered organic carbon in soils, especially for the carbon sequestration driven by tree roots (Hoover and Heath, 2015; Popkin, 2019). Some studies have shown that soil microbes play a key role in the belowground carbon cycle of forest ecosystems in China (Zhou and Wang, 2015; Qi and Yang, 2017); however, others have suggested that soil microorganisms do not play a major role (Sierra et al., 2015; Wei et al., 2015). Tree species and microbial diversity also affect soil carbon sequestration in forests (Bardgett and van der Putten, 2014; Deng et al., 2016; Khlifa et al., 2017). Thus, identifying and quantifying the relative influence of root-microbial systems in forests remain a challenge that should be addressed in future studies (Laganière et al., 2017).

Isotope techniques have been widely used to investigate the processes occurring at the soil–root interface (Rugova et al., 2017). However, most rhizosphere studies have used isotope labeling methods in the laboratory, and studies of natural isotopes in the field are rare (Haichar et al., 2016). The stable isotope method is a feasible approach for rhizosphere studies of forest SOC sequestration (Gautam and Lee, 2016). In order to better use the stable isotope indicators, the natural fractionation of stable isotopes should be understood, but the stable isotope variation following soil carbon sequestration in a root–microbial system is still not fully understood (Gautam and Lee, 2016; Haichar et al., 2016).

In this field study, soil microbial community of degraded farm land and climax nature forest was measured. The experimental devices were used to simulate the different primary stage of succession. We focused on variations in the microbial community and N isotope levels caused by the processes of tree roots sequestrating SOC. The aims of the current study were to determine how the diversity, ^15^N, and species interactions of the active rhizomicrobial community changed during soil carbon sequestration and examine the interactions driving these changes.

## Materials and Methods

### Experimental Set-Up and Sampling

**FIGURE 1:**
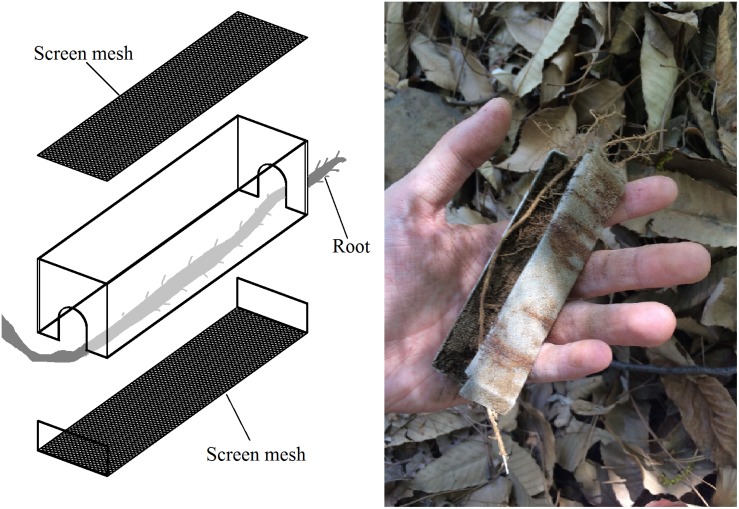
Experimental device for sampling.

This experimental site was established at Jiufeng National Forest Park, which is located in Beijing, China (40.06°N, 116.09°E). Experimental devices were 2 × 2 × 10 cm stainless steel boxes with 800-screen mesh at the top and bottom such that soil gas and water could pass through the box without any fine soil particles (Figure 1). Tree roots (about 2–5 mm in diameter) were passed through the boxes (Figure 1). The boxes were filled with homogeneous control soil (CS) from an abandon farmland, with known soil properties. The experimental groups were as follows: the middle of *Pinus tabuliformis* Carrière roots (MPR group); the tips of the same *P. tabuliformis* roots (TPR group); the middle of *Quercus variabilis Bl.* roots (MQR group); the tips of the same *Q. variabilis* roots (TQR group); post-fire *P. tabuliformis* roots (FPR group); and dead *P. tabuliformis* roots (DPR group). We also collected soil samples from a natural *P. tabuliformis* forest (NFS) which is a climax community in this area. Each group included five replicates. After 1 year, the boxes were removed, and the samples were subjected to physical property, molecular, and bioinformatic analyses.

### Stable Isotope Analysis

Soil samples were cleaned to remove roots and stones before being ground to grain sizes of <0.25 mm. Soil and root samples were analyzed to obtain their δ^13^C, δ^15^N, C, and N contents using a DELTA V Advantage Isotope Ratio Mass Spectrometer (Flash EA1112 HT Elemental Analyzer; Thermo Fisher Scientific Inc., United States). The measurement precisions for δ^13^C and δ^15^N were ±<0.1 and ±<0.2‰, respectively.

^13^C fractionation between roots and SOC (*F*_*SOC*_) was calculated from the isotopic differences between the SOC convert from roots and tree roots; the isotopic compositions of the newly sequestered SOC (δ^13^*C*_*NSC*_) was calculated from root values when partitioning soil organic matter as:

(1) $$\delta^{13}\,C_{N\,S\,C}=\delta^{13}\,C_{R}+F_{S\,O\,C},$$

where δ^13^*C*_*R*_ is the root δ^13^*C* value (Song et al., 2017).

The contributions of old (CS) and newly sequestered SOC (NSC) sources were calculated using linear two-source isotopic mixing models, as follows:

(2) $$f_{N\,S\,C}=\left(\delta^{13}\,C_{T}-\delta^{13}\,C_{C\,S}\right)/\left(\delta^{13}\,C_{N\,S\,C}-\delta^{13}\,C_{C\,S}\right),$$

(3) $$f_{C\,S}=1-f_{N\,S\,C},$$

(4) $$C_{T}=C_{N\,S\,C}+C_{C\,S},$$

(5) $$\text{and}\,C_{N\,S\,C}=C_{T}\cdot f_{N\,S\,C}$$

where δ^13^*C*_*T*_ is the isotopic composition of total SOC; *C*_*T*_ is the total SOC content; δ^13^*C*_*NSC*_ and δ^13^*C*_*CS*_ are the isotopic compositions of the newly sequestered SOC and CS sources, respectively; *C*_*NSC*_ and *C*_*CS*_ are the total SOC content of newly sequestered SOC and CS sources, respectively; and *f*_*NSC*_ and *f*_*CS*_ are the proportional contributions of newly sequestered SOC and CS sources to total SOC, respectively (Song et al., 2017).

### Molecular and Bioinformatic Analyses

Genomic DNA was extracted from 0.5 g fresh soil samples using a PowerSoil DNA Isolation Kit (MoBio Laboratories, Carlsbad, CA, United States) following the manufacturer’s instructions. All extracted DNA samples were stored at −20°C for subsequent analysis. To assess the bacterial and fungal community compositions, we amplified the V3–V4 hypervariable region of the bacterial 16S rRNA gene using the forward primer 338F (5′-ACTCCTACGGGAGGCAGCAG-3′) and the reverse primer 806R (5′-GGACTACHVGGGTWTCTAAT-3′) and the fungal ITS region using the forward primer ITS1-F (5′-CTTGGTCATTTAGAGGAAGTAA-3′) and the reverse primer ITS2 (5′-TGCGTTCTTCATCGATGC-3′). These primers contained a set of 8-nucleotide barcode sequences unique to each sample. Polymerase chain reaction (PCR) amplifications were performed following the procedure described by Taş et al. (2014). PCR products were pooled and purified using a QIAquick Gel Extraction Kit (Qiagen, Germany). The purified PCR products were pooled at equimolar concentrations and paired-end sequenced (2 × 300) on an Illumina MiSeq platform according to the standard protocols.

The raw data were screened, and sequences were removed from consideration if they were shorter than 200 bp, had a low quality score (≤20), contained ambiguous bases, or did not exactly match to primer sequences and barcode tags. We processed the high-quality sequence data in the QIIME package (Quantitative insights into microbial ecology; v1.2.1), according to the procedure described by Caporaso et al. (2012). The unique sequence set was classified into operational taxonomic units (OTUs) based on the threshold of 97% identity using UCLUST. Chimeric sequences were identified and removed using Usearch (version 8.0.1623). The taxonomy of each 16S rRNA gene sequence was analyzed against the Silva119 16S rRNA database using UCLUST with a confidence threshold of 97%, and the taxonomy of each ITS gene sequence was analyzed by comparison against sequences within the Unite 7.0 database using UCLUST. The complete dataset was sent to the Sequence Read Archive (SRA) database of the National Center for Biotechnology Information (NCBI) under the accession numbers of PRJNA587579.

### Statistical Analysis

Taxonomic alpha diversity, which is the diversity of the microbial communities based on individual samples (within-habitat diversity), was calculated according to the Shannon index using Mothur software (v.1.30.1). The difference in the amount of reads per sample was standardized using the percentage of total reads/OTUs in a sample (McMurdie and Holmes, 2014). The unweighted and weighted UniFrac distances (beta diversity) were visualized by principle coordinate analysis (PCoA) plots and shown as distance-heatmaps (Jami et al., 2013). Analysis of similarities within the vegan package in R (999 permutations) was performed to test the significance of separation. FAPROTAX and FUNGuild were used for the functional annotation of 16S and ITS taxa (Louca et al., 2016; Nguyen et al., 2016).

The values presented in the figures are given as means ± standard errors of means. The R package for Bayesian isotopic mixing models was used to estimating and reducing the uncertainty as described by Parnell et al. (2010). Statistical analyses were performed with IBM SPSS statistics 23.0 (IBM Inc., NY, United States).

## Results

### Changes in Soil C%, N%, and Bacterial and Fungal Community Diversity

Compared with the CS group, C% and N% were increased in all other groups, except FPR (Supplementary Figure S1A). The C/N were also changed compared with the CS group (Supplementary Figure S1B). Thus, the C% and N% were greatly affected by tree roots (Song and Liu, 2018).

**FIGURE 2:**
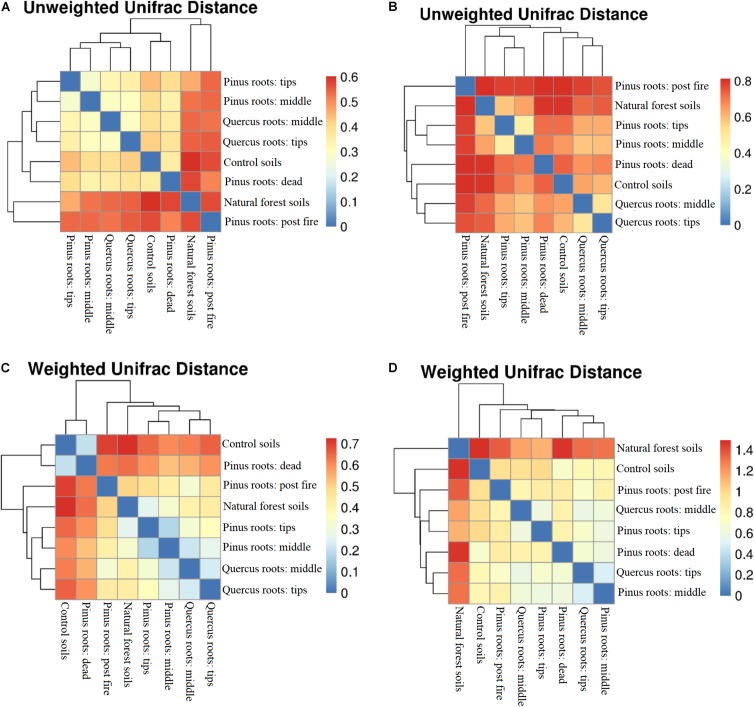
Distance-heatmaps of soil unweighted **(A)** bacterial and **(B)** fungal UniFrac distances and weighted **(C)** bacterial and **(D)** fungal UniFrac distances.

An OTU-level approach was performed to calculate soil bacterial and fungal alpha diversity (Shannon index; Supplementary Figure S2). Compared with the Shannon index in the CS group, only the DPR and FPR groups showed lower values with respect to bacterial diversity, and all groups showed increased fungal diversity (Supplementary Figure S2). Soil bacterial and fungal beta diversity (unweighted and weighted UniFrac distances) are shown as distance-heatmaps (Figure 2). We found that CS was closer to DPR, TPR was closer to MPR, TQR was closer to MQR, and NFS was closer to FPR in both unweighted and weighted bacterial distance-heatmaps (Figures 2A,C). In contrast, NFS was not close to FPR in the unweighted fungal distance-heatmap (Figure 2B). The groups with living roots were closer to each other than other groups, and NFS was far away from the other groups in the weighted fungal distance-heatmap (Figure 2D).

### Changes in Soil Bacterial and Fungal Community Compositions

**FIGURE 3:**
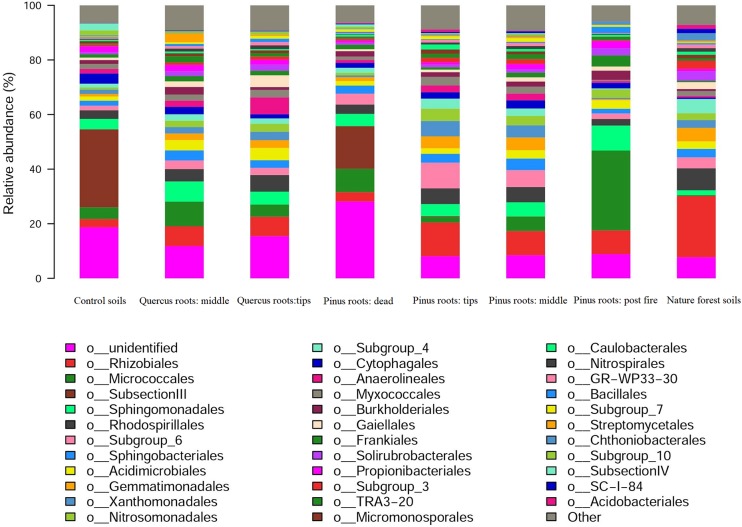
Distribution of 16S rRNA sequences across bacterial communities.

The dominant bacterial orders of each group were as follows: *SubsectionIII* in CS; *Micrococcales*, *Rhizobiales*, and *Sphingomonadales* in MQR; *Rhizobiales* and *Rhodospirillales* in TQR; *SubsectionIII* in DPR; *Rhizobiales* in TPR; *Rhizobiales* in MPR; *Micrococcales* in FPR; and *Rhizobiales* in NFS (Figure 3). Notably, *Rhizobiales* showed significantly high abundance in live roots in the MQR, TQR, TPR, MPR, and NFS groups, and *SubsectionIII* showed significantly high abundance in the CS and DPR groups (Figure 3). *Micrococcales* had highest abundance in the FPR group, followed by *Rhizobiales* and *Sphingomonadales* (Figure 3).

**FIGURE 4:**
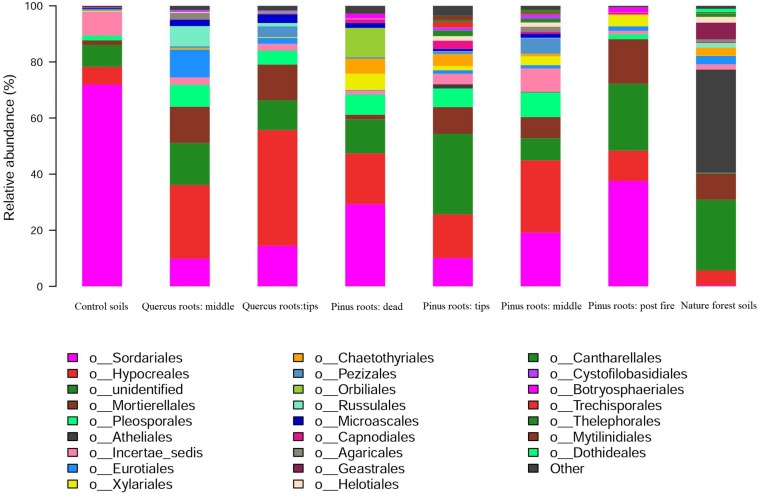
Distribution of ITS sequences across fungal communities.

The dominant fungal orders of each group were as follows: *Sordariales* in CS, *Hypocreales* in MQR, *Hypocreales* in TQR, *Sordariales* and *Hypocreales* in DPR, *Hypocreales* in TPR, *Hypocreales* and *Sordariales* in MPR, *Sordariales* in FPR, and Atheliales in NFS (Figure 4). *Sordariales* and *Hypocreales* showed significantly high abundance in all groups except the NFS group (Figure 4). Furthermore, the abundance of *Hypocreales* was higher in the MQR, TQR, TPR, MPR, and NFS groups, whereas that of *Sordariales* was higher in the CS and DPR groups (Figure 4). However, the abundances of *Sordariales* and *Hypocreales* were very low in the NFS group (Figure 4). The abundance of Russulales was higher in the MQR group than in the TQR group (Figure 4).

### Changes in Newly Sequestered SOC

**FIGURE 5:**
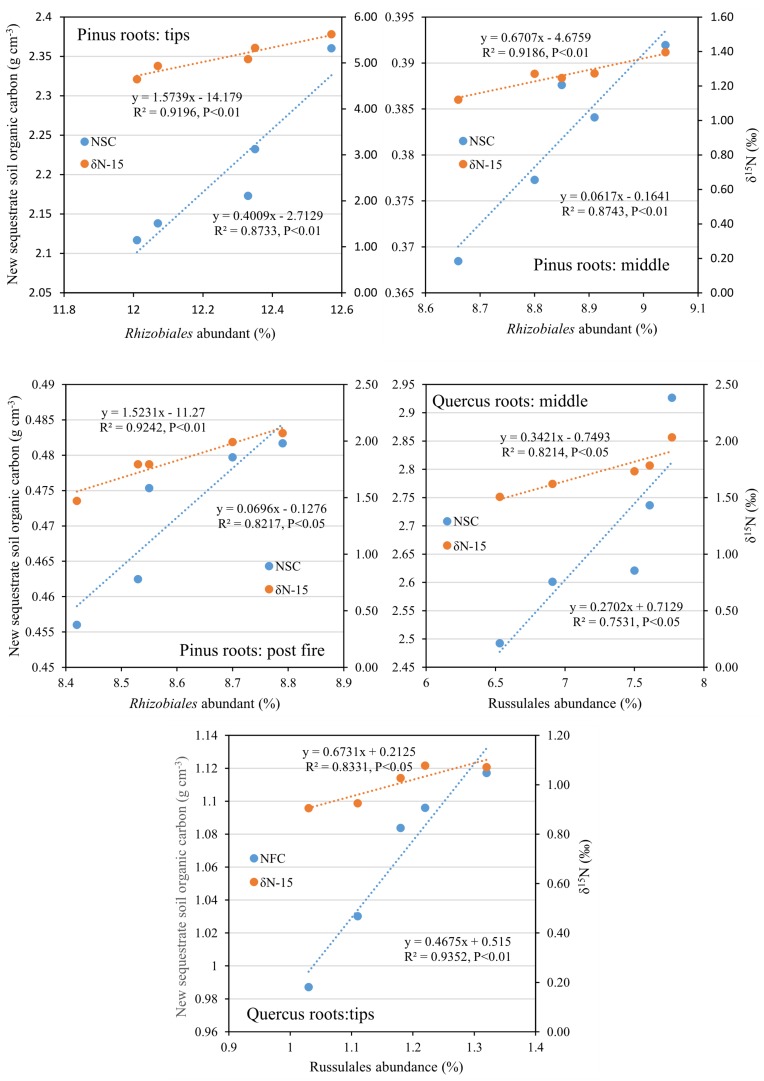
Relationship between newly sequestered SOC (NSC) or δ^15^N (δN-15) and bacterial order *Rhizobiales* in middle portion of *Pinus tabuliformis* roots; tips of *P. tabuliformis* roots; and post-fire planted forest of *P. tabuliformis.* Relationship between newly sequestered SOC (NSC) or δ^15^N (δN-15) and fungi order Russulales in middle portion of *Quercus variabilis* roots; and tips of *Q. variabilis* roots.

**FIGURE 6:**
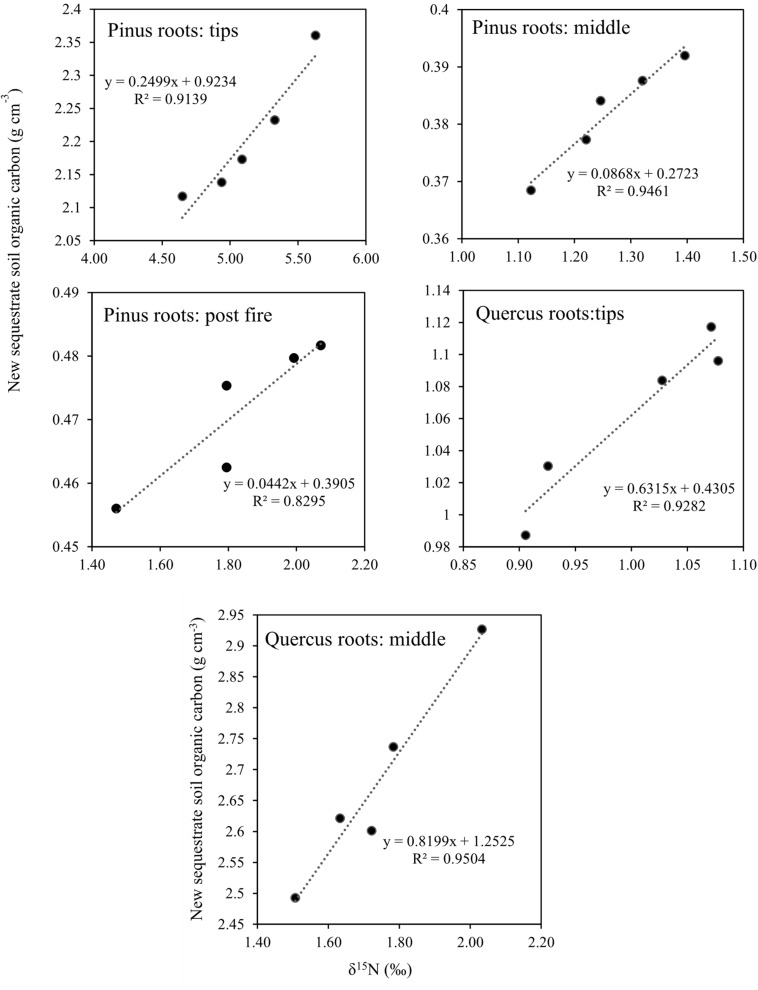
Relationship between newly sequestered SOC and δ^15^N in middle portion of *Pinus tabuliformis* roots; tips of *P. tabuliformis* roots; post-fire planted forest of *P. tabuliformis.* Relationship between newly sequestered SOC and δ^15^N in middle portion of *Quercus variabilis* roots; and tips of *Q. variabilis* roots.

**FIGURE 7:**
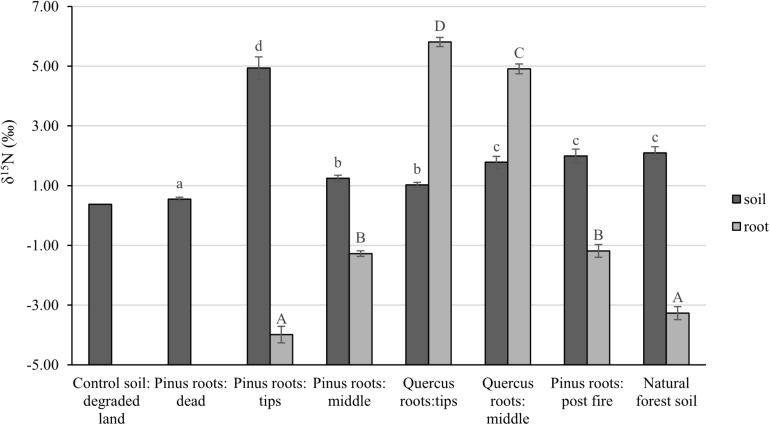
The δ^15^N values of soil and roots of control soil; middle portion of *Pinus tabuliformis* roots; tips of *P. tabuliformis* roots; middle portion of *Quercus variabilis* roots; tips of *Q. variabilis* roots; post-fire planted forest of *P. tabuliformis*; dead *P. tabuliformis* roots; and natural *P. tabuliformis* forest. Different letters indicate significant differences at *P* < 0.05.

After 1 year, the contents of newly sequestered SOC are shown in Supplementary Figure S3. The abundance of the bacterial order *Rhizobiales* was significantly positively correlated with newly sequestered SOC and δ^15^N of *P. tabuliformis* live root-affected groups (TPR, MPR, and FPR; Figure 5). The abundance of the fungal order Russulales was positively correlated with newly sequestered SOC and δ^15^N of the *Q. variabilis* live root-affected groups significantly (TQR and MQR; Figure 5). Furthermore, the δ^15^N values of the *P. tabuliformis* live root-affected groups (TPR, MPR, and FPR) were positively correlated with newly sequestered SOC significantly (Figure 6), and the δ^15^N values of *Q. variabilis* live root-affected groups (TPR, MPR, and FPR) were also significantly positively correlated with newly sequestered SOC (Figure 6). The δ^15^N values of soil and roots in each group are shown in Figure 7.

## Discussion

### Influence of Tree Roots on Soil Microbial Diversity and Community Composition

According to the experimental design, the CS was from a degraded abandon farmland, so CS and DPR groups appeared to reflect the status of the degraded ecosystem; the tree roots of TPR, MPR, TQR, MQR, and FPR had been living in degraded soil for a year, they were in the primary stage of succession; and NFS was in a climax community (Song and Liu, 2018). In degraded ecosystems (CS and DPR), decay processes predominate, and only some autotrophic or saprophytic microorganisms can survive (López-ballesteros et al., 2018; Song and Liu, 2018), thus resulting in low Shannon indexes and *SubsectionIII* becoming the dominant bacterial order. Furthermore, the dead root decomposition alters microbial enzyme dynamics, making the sources of nutrition more diverse and increasing bacterial diversity (Nannipieri et al., 2003; Rinkes et al., 2016). Therefore, the bacterial Shannon index of the DPR group was larger than that of the CS group significantly (*P* < 0.05). Tree roots contain rhizo-microbes (such as *Rhizobiales*) during primary succession and establish a suitable environment for facultative bacteria (such as *Micrococcales*, *Sphingomonadales*, and *Rhodospirillales*) (Elhottová et al., 2009; Tanaka and Nara, 2009). Such mechanisms of coexistence resulted in higher bacterial Shannon indexes for the TPR, MPR, TQR, and MQR groups. Finally, rhizo-microbes became the dominant population in the climax community, and some types of bacteria were eliminated during the succession; the components of *Rhizobiales* were large, and bacterial diversity decreased in the NFS group.

In contrast to bacterial diversity, the fungal diversity of the DPR group was not significantly higher than that of the CS group (*P* > 0.05), and the fungal diversities of the TPR, MPR, TQR, and MQR groups were not higher than that of the NFS group (*P* > 0.05). Typically, saprotrophic fungi are less effective than bacteria during the decay process of abandoned land (Ren et al., 2017; López-ballesteros et al., 2018); thus, the fungal diversity of the DPR group was not much higher than that of the CS group. However, when roots bring exudates into soil, the fungi of the rhizosphere will become more and more important in nutrient cycling and ecological succession; therefore, the fungal diversity of the NFS group was still high, even though some species were eliminated (Hannula et al., 2017). Furthermore, the composition of the active functional fungal community changed from one composed of fast-growing and pathogenic fungal species (such as *Hypocreales*) to one consisting of beneficial and slower-growing mycorrhiza fungal species (such as Atheliales) (Hannula et al., 2017; Song and Liu, 2018), which may have consequences for dominant fungal order shifts from *Sordariales* (degraded ecosystem) to *Hypocreales* (primary succession) and finally Atheliales (climax community).

Both soil bacterial unweighted and weighted UniFrac distances did not show relationships among groups. Importantly, the bacterial unweighted and weighted UniFrac distances between the CS and FPR groups and between the CS and NFS groups were similar. Forest fires had burned much of the soil organic matter, such that the C% and N% of the FPR group were the lowest of all groups. Changes in post-fire soil nutrients made the bacterial Shannon index of the FPR group higher than that of the CS group (*P* < 0.05) (Prendergast-Miller et al., 2017; Rodríguez et al., 2017), but was difficult to use for soil microbes due to the presence of pyrogenic organic matter (Soong et al., 2017). The bacterial Shannon index of the FPR group was still lower than those of the TPR, MPR, TQR, MQR, and DPR groups (*P* < 0.05). After recovery of the soil microbial community, the dominant species had been shifted from fast-growing, pathogenic bacteria to beneficial, slower-growing fungal species, and the bacterial populations were eliminated (Chang et al., 2017; Hannula et al., 2017); thus, the bacterial diversity and UniFrac distances of the NFS group were similar to those of the FPR group. Compared with the bacterial community, the fungal community changed slower and more significantly (Hannula et al., 2017; Song and Liu, 2018), and therefore, the fungal unweighted UniFrac distances between each group were further than those in the bacterial heatmap. Furthermore, fungal biomass was significantly increased and was not eliminated like bacteria during the entire succession process (Deng et al., 2016). Because of this, the distances of primary succession (TPR, MPR, TQR, MQR, and FPR) were closer between each other than between degraded ecosystems (CS and DPR), and the distance of the climax community (NFS) was far away from other groups in the weighted fungal distance-heatmap. Therefore, the fungal unweighted UniFrac distances could well show the different microbial species structures, and the fungal weighted UniFrac distances could well show the difference of different successive stages.

### Correlation of Newly Sequestered SOC With Microbial Community Composition and δ^15^N

Compared with the CS group, C% and N% were increased in all groups except the FPR group. Thus, the tree root provided benefits of soil carbon stock, and the burned SOC required a long recovery time. The roots of *P. tabuliformis* and *Q. variabilis* showed different strategies for carbon sequestration. The root-driven newly sequestered SOC of *P. tabuliformis* was concentrated at the apical region, whereas that of *Q. variabilis* was concentrated at the middle region. Therefore, we believe that *P. tabuliformis* preferred to concentrate root exudates to the tip and stimulate rhizo-microbes such that the roots could grow longer to obtain nutrients and water (Song and Liu, 2018). However, *Q. variabilis* preferred to concentrate root exudates to the middle region and stimulate arbuscular mycorrhizal fungi (such as Russulales) colonizing to tree roots, such vesicular-arbuscular mycorrhizal can help host plant to obtain nutrients and expanded the absorption surface (Dickie et al., 2001; Bever et al., 2010). In this way, *Q. variabilis* could use most of the length of the root, which may be helpful to adapt poor, shallow soils (Song and Liu, 2018).

Evidences for δ^15^N indicated that the carbon sequestration processes of *P. tabuliformis* and *Q. variabilis* roots were different. Unlike other bacterial orders, most OTUs of *Rhizobiales* in *P. tabuliformis* forests were found to be rhizomicrobes (chemoheterotrophy or nitrogen fixation bacteria), not saprophytic bacteria or pathogens (Table 1). *P. tabuliformis* concentrates carbon-rich root exudates to the tip and stimulates bacteria (particularly *Rhizobiales*) to exchange the nitrogen from soil (Kuzyakov and Xu, 2013; Song et al., 2017), resulting in a high C/N ratio for TPR. Additionally, the rhizomicrobes supply relatively ^15^N-depleted N to their hosts in the denitrification and nitrogen fixation (Högberg et al., 1996); thus, the δ^15^N of roots became very low in *P. tabuliformis* forests. *Rhizobiales* also could help mycorrhizal supply nourishment for plants, make mycorrhizal healthier and more active, and increased the ability of root-driven carbon sequestration (Nguyen and Bruns, 2015; Zavattieri et al., 2016; Garcia-Lemos et al., 2019; Wagner et al., 2019). Therefore, the abundance of the bacterial order *Rhizobiales* was positively correlated with newly sequestered SOC and δ^15^N from the *P. tabuliformis* live root-affected groups, suggesting that *Rhizobiales* was the dominant order of root-driven carbon sequestration in *P. tabuliformis* forests.

**TABLE 1:** The relative abundance and functional annotation of bacterial order *Rhizobiales* in the *Pinus tabuliformis* forests.

Group	TPR	MPR	FPR	NFS
Chemoheterotrophy	0.052060767	0.038833957	0.04202105	0.0893958
Aerobic_chemoheterotrophy	0.051958667	0.038754857	0.04178143	0.0893743
Nitrogen_fixation	0.022486826	0.011456564	0.004513681	0.056790601
Photoheterotrophy	0.013602413	0.00505669	0.006790493	0.015980373
Phototrophy	0.013602413	0.00505669	0.006790493	0.015980373
Nitrous_oxide_denitrification	0.013173481	0.0047604	0.008068704	0.015658225
Denitrification	0.013173481	0.0047604	0.008068704	0.015658225
Nitrate_respiration	0.013173481	0.0047604	0.008068704	0.015658225
Nitrate_reduction	0.013173481	0.0047604	0.008068704	0.015658225
Nitrogen_respiration	0.013173481	0.0047604	0.008068704	0.015658225
Nitrate_denitrification	0.012765001	0.004088808	0.006790493	0.015421955
Nitrite_denitrification	0.012765001	0.004088808	0.006790493	0.015421955
Nitrite_respiration	0.012765001	0.004088808	0.006790493	0.015421955
Anoxygenic_photoautotrophy_S_oxidizing	0.012765001	0.004088808	0.006790493	0.015421955
Anoxygenic_ photoautotrophy	0.012765001	0.004088808	0.006790493	0.015421955
Photoautotrophy	0.012765001	0.004088808	0.006790493	0.015421955
Dark_oxidation_of_sulfur_compounds	0.000653596	0.001580216	0.001837428	0

*MPR, middle portion of *Pinus tabuliformis* roots; TPR, tips of *P. tabuliformis* roots; FPR, post-fire planted forest of *P. tabuliformis* NFS, natural *P. tabuliformis* forest.*

However, there were no significant differences between the *Rhizobiales* compositions of the TQR and MQR groups, and the newly sequestered SOC of the MQR group was larger than that of TQR. In addition, the δ^15^N of roots was higher than that in soils in the TQR and MQR groups, but the C/N did not show any significant differences between the TQR and MQR groups. The abundance of the fungal order Russulales (all OTUs were symbiotroph mycorrhiza fungi; Table 2) was positively correlated with the newly sequestered SOC and δ^15^N of the *Q. variabilis* live root-affected groups, although the composition of Russulales was much lower than that of *Hypocreales*. These results showed that the root-driven carbon of *Q. variabilis* was not beneficial to the fast-growing bacteria and fungi, which mainly used root exudates for rapid growth and decomposition of soil organic matter (Yin et al., 2016). However, some minority mycorrhiza fungi (such as Russulales) promote the retention and stabilization of microbial-derived organic matter (Kayler et al., 2011; Hannula et al., 2017), resulting in enrichment of ^15^N in stabilized organic matter (Conen et al., 2007) and transport of ^15^N-enriched N to their hosts; this process contributes to carbon sequestration (Angst et al., 2019).

**TABLE 2:** The relative abundance and functional annotation of fungal order Atheliales and Russulales of the MQR, TQR, and NFS group.

ID	MQR	TQR	NFS	Order	Taxon	Trophic mode	Guild	Confidence ranking
1	0.071577	0.011799	0	Russulales	Russula	Symbiotroph	Ectomycorrhizal	Highly probable
2	0	0	0.015843	Russulales	Russulaceae	Symbiotroph	Ectomycorrhizal	Probable
3	5.41*E*−05	0	0.367322	Atheliales	Amphinema	Symbiotroph	Ectomycorrhizal	Highly probable
4	0	0	0.000386	Atheliales	Athelopsis	Saprotroph	Undefined Saprotroph	Probable
5	0	0	3.68*E*−05	Atheliales	Athelopsis	Saprotroph	Undefined Saprotroph	Probable
6	0	0	5.51*E*−05	Atheliales	Amphinema	Symbiotroph	Ectomycorrhizal	Highly probable

*MQR, middle portion of *Quercus variabilis* roots; TQR, tips of *Q. variabilis* roots; NFS, natural *P. tabuliformis* forest.*

Soil δ^15^N may reflect the degree to which soil organic matter has been enriched in ^15^N by microbial processing and sequestered SOC at the same time (Craine et al., 2015b). The soil δ^15^N increases with increasing microbial processing also evidenced by greater δ^15^N of high C/N fractions (Song et al., 2017). However, unlike the live root-affected groups, soils of DPR have a lower C/N due to the soil δ^15^N increases with decreasing C/N during litter decomposition processes (Craine et al., 2015a). Thus, the organic matter from all groups has undergone a different pathway to carbon sequestration, and the organic matter of the live root-affected groups may highly protected within soil micro-structures (Kayler et al., 2011). Nevertheless, regardless of the process, the δ^15^N values of both *P. tabuliformis* and *Q. variabilis* root-driven organic matter increased and were positively correlated with newly sequestered SOC. Therefore, δ^15^N could be an important indicator to estimate root-driven carbon sequestration.

Root-driven carbon sequestration may mainly be attributable to the exchange and transport of nutrients between tree roots and bacteria (such as *Rhizobiales*) or fungi (such as Russulales) in the rhizosphere during primary succession. The bacterial order *Rhizobiales* played roles as a “nutrition exchanger” (which can exchange nutrition between root and soil), representing a major factor in *P. tabuliformis* root-driven carbon sequestration. The fungal order Russulales played roles as a “stability promoter” (which can promote the retention and stabilization of microbial-derived organic matter) and “nutrition transporter” (which can transport nutrition between root and soil), representing a major factor in *Q. variabilis* root-driven carbon sequestration. However, regardless of the process, the δ^15^N values of both *P. tabuliformis* and *Q. variabilis* root-driven organic matter were increased and positively correlated with newly sequestered SOC. Therefore, δ^15^N could be an important indicator to estimate root-driven carbon sequestration. Compared with previous studies, we believe that the dominant rules of *Rhizobiales* and Russulales in carbon sequestration could be replaced by other fungi that were more able to adapt to the rhizosphere environment during ecological succession (Hannula et al., 2017; Kolaříková et al., 2017; Song and Liu, 2018). Thus, very large C% and N% were observed as Atheliales (most OTUs were symbiotroph mycorrhiza fungi; Table 2) became the dominant order in the NFS group.

## Data Availability Statement

The datasets generated for this study can be found in the National Center for Biotechnology Information (NCBI): PRJNA587579.

## Author Contributions

WS, XT, and YL developed the ideas and designed the experimental plans. WS performed the experiments, analyzed the data, and wrote the manuscript. WL made the tables.

## Conflict of Interest

The authors declare that the research was conducted in the absence of any commercial or financial relationships that could be construed as a potential conflict of interest.

## Supplementary Material

FIGURE S1 **(A)** C% and N%, **(B)** C/N ratio of control soil; middle portion of *Pinus tabuliformis* roots; tips of *P. tabuliformis* roots; middle portion of *Quercus variabilis* roots; tips of *Q. variabilis* roots; post-fire planted forest of *P. tabuliformis*; dead *P. tabuliformis* roots; and natural *P. tabuliformis* forest. Different letters indicate significant differences at *P* < 0.05.

FIGURE S2 Soil bacterial and fungal alpha diversity (Shannon index, *H*) of control soil; middle portion of *Pinus tabuliformis* roots; tips of *P. tabuliformis* roots; middle portion of *Quercus variabilis* roots; tips of *Q. variabilis* roots; post-fire planted forest of *P. tabuliformis*; dead *P. tabuliformis* roots; and natural *P. tabuliformis* forest. Different letters indicate significant differences at *P* < 0.05.

FIGURE S3 Newly sequestered SOC after 1 year in middle portion of *Pinus tabuliformis* roots; tips of *P. tabuliformis* roots; middle portion of *Quercus variabilis* roots; tips of *Q. variabilis* roots; and post-fire planted forest of *P. tabuliformis*. Different letters indicate significant differences at *P* < 0.05.
