# Supplementary figures and images for: Microbial Community, Newly Sequestered Soil Organic Carbon, and δ15N Variations Driven by Tree Roots
Source: Front Microbiol. 2020 Feb 27;11:314. doi: 10.3389/fmicb.2020.00314 (PMC7056912; doi:10.3389/fmicb.2020.00314)

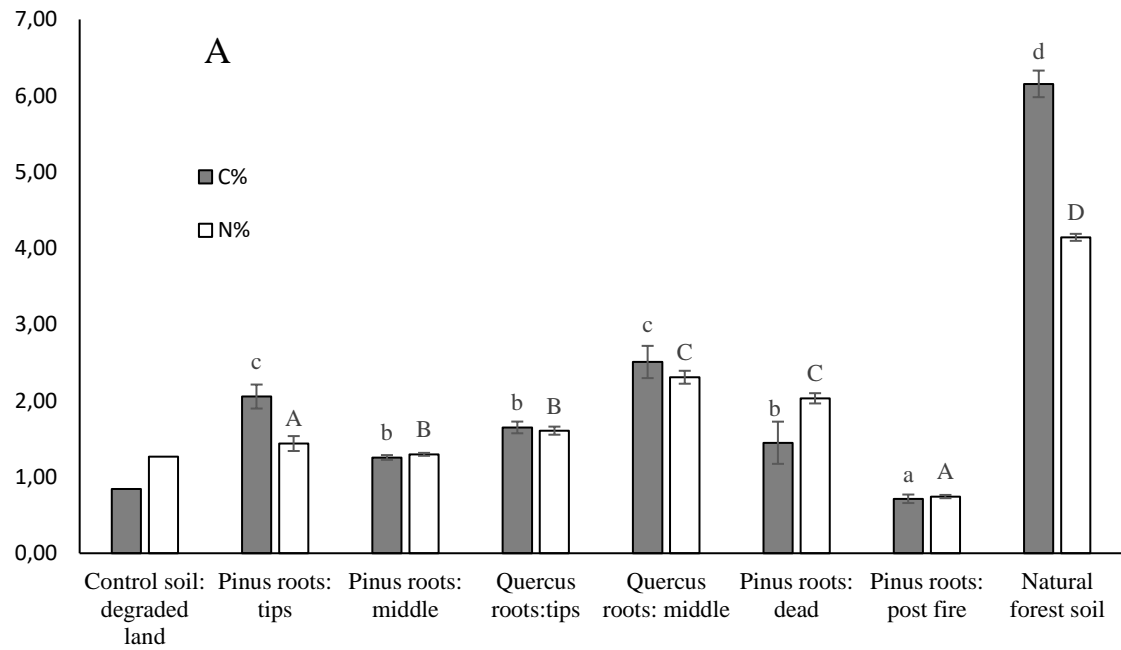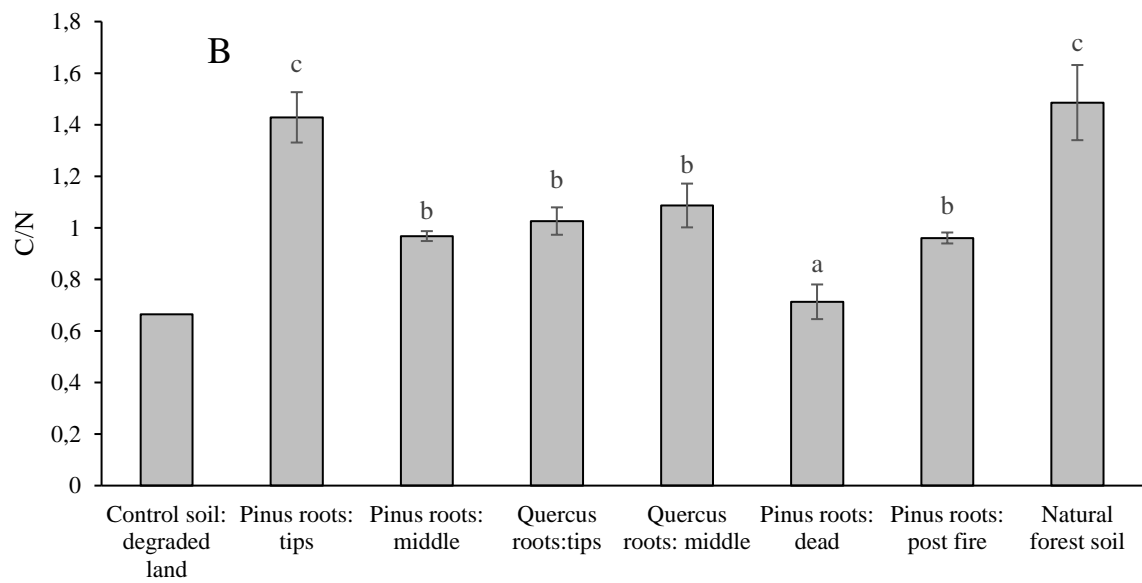

Supplement: FIGURE S1 — (A) C% and N%, (B) C/N ratio of control soil; middle portion of Pinus tabuliformis roots; tips of P. tabuliformis roots; middle portion of Quercus variabilis roots; tips of Q. variabilis roots; post-fire planted forest of P. tabuliformis; dead P. tabuliformis roots; and natural P. tabuliformis forest. Different letters indicate significant differences at P < 0.05. [file Image_1.pdf]

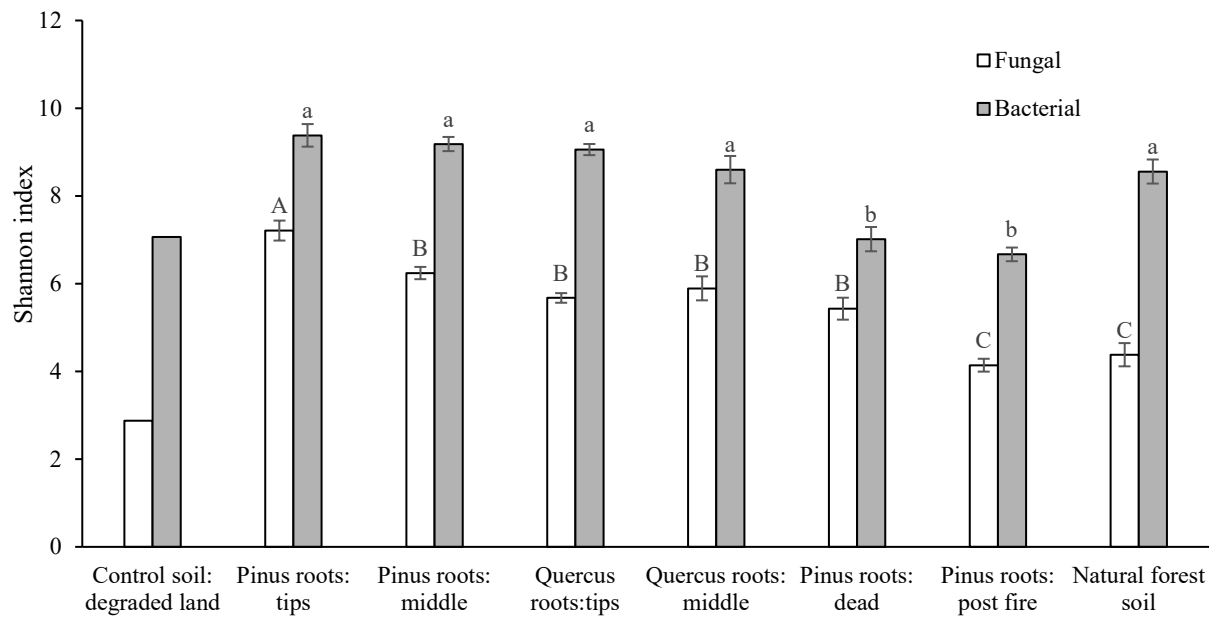

Supplement: FIGURE S2 — Soil bacterial and fungal alpha diversity (Shannon index, H) of control soil; middle portion of Pinus tabuliformis roots; tips of P. tabuliformis roots; middle portion of Quercus variabilis roots; tips of Q. variabilis roots; post-fire planted forest of P. tabuliformis; dead P. tabuliformis roots; and natural P. tabuliformis forest. Different letters indicate significant differences at P < 0.05. [file Image_2.pdf]

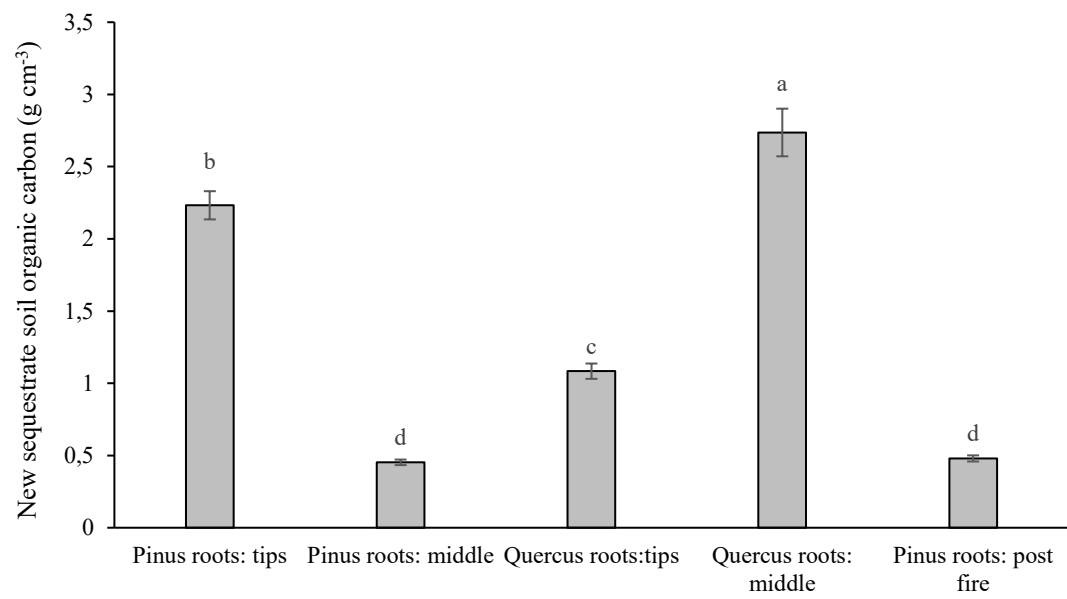

Supplement: FIGURE S3 — Newly sequestered SOC after 1 year in middle portion of Pinus tabuliformis roots; tips of P. tabuliformis roots; middle portion of Quercus variabilis roots; tips of Q. variabilis roots; and post-fire planted forest of P. tabuliformis. Different letters indicate significant differences at P < 0.05. [file Image_3.pdf]
